# Pain Intensity of Skeletally Anchored Maxillary Molar Distalization in Conjunction with Micro-osteoperforations: A Randomized Clinical Trial

**DOI:** 10.7759/cureus.53527

**Published:** 2024-02-03

**Authors:** Abdallah Mohammed Bahaa El-Din, Khaled Abd El Khaliq Hendy, Raafat Elghetany Mohamed, Ahmed Abouelnour, Mohamed Mohamed Ali, Ahmed Akram El-Awady, Farouk Ahmed Hussein

**Affiliations:** 1 Department of Orthodontics, Faculty of Dental Medicine (Boys), Al-Azhar University, Cairo, EGY

**Keywords:** class ii malocclusion, molar distalization, distal jet, pain, micro-osteoperforation

## Abstract

Objective

To assess pain intensity levels during orthodontic therapy of Class II malocclusion patients undergoing skeletally anchored maxillary molar distalization assisted with different micro-osteoperforation (MOP) approaches.

Methods

Twenty-seven patients (12 males and 18 females) with a mean age of 16.1 ± 0.3 years were randomized into three equal groups (n=9): Group 1 comprised MOPs on buccal surface, Group 2 comprised MOPs on buccal and palatal surface, and Group 3 comprised the control or no-MOP group. The patients underwent maxillary molar distalization using skeletally anchored distal jet appliance assisted with or without MOPs. The MOPs were applied repeatedly on the buccal and buccal and palatal sides, or no MOP (control). Pain intensity was assessed using a 10 cm visual analog scale after each device activation at 24, 48, 72 hours, and at seven days. Data were analyzed using one-way ANOVA and repeated measures ANOVA for non-paired and paired means.

Results

Both approaches of buccal and buccal and palatal application of MOPs showed statistically significant (p< 0.01) higher levels of pain intensity after the first activation at 24 hours. Nevertheless, pain intensity levels decreased significantly in both MOP groups and between the two activations.

Conclusion

The repeated application of MOPs on either the buccal side only or on both buccal and palatal sides during maxillary molar distalization did not affect the levels of pain experienced; however, these levels were reported to be higher than that obtained in the control group. Moreover, it is observed that these pain levels tend to gradually reduce to mild levels over the subsequent days.

## Introduction

Molar distalization is a commonly used treatment modality for correcting Class II malocclusion in the maxillary arch. This treatment establishes a Class I molar and canine relationship, which can be achieved through extraoral or intraoral appliances [1,2]. Occasionally, orthodontic mechanics can lead to unwanted tooth movement, including buccal displacement of molars and proclination of incisors. To overcome these drawbacks, temporary skeletal anchorage devices are utilized to support intraoral distalization appliances, which can help maintain the proper alignment of teeth [2,3].

On average, fixed orthodontic treatment takes around 20 months but can extend up to 33 months [4,5]. However, patients generally expect a much shorter duration of treatment. Therefore, one of the leading research areas in modern orthodontics is exploring ways to shorten treatment time. This is particularly important for adult patients who may avoid orthodontic treatment due to the lengthy treatment period [6,7].

The methods for accelerating tooth movement rely on stimulating the biological tissue reaction. These methods can be categorized into two categories based on the degree of invasiveness: conservative (biological and physical) and surgical treatments [8,9]. The use of pharmaceuticals, such as prostaglandins, vitamin D, beta 2-adrenergic agonist, thyroxine, growth hormone, parathyroid hormone, osteocalcin, and receptor activator of nuclear factor kappa-B ligand/receptor activator of nuclear factor kappa-Β protein, to increase tooth movement in animal experiments and human subjects are the conservative-biological methods. The conservative-physical approaches, which rely on the use of device-assisted therapy, include low-level laser therapy, vibration, photobiomodulation, pulsed electromagnetic field, direct electric currents, and photobiomodulation. After validation, these approaches have demonstrated encouraging outcomes [8].

With the potential to drastically shorten the course of treatment, surgical techniques are thought to be the most clinically utilized and extensively evaluated [8]. They do, however, rely on the "regional acceleratory phenomenon (RAP)," since the orthodontic tooth movement may be momentarily accelerated in cases where surgical injury to the alveolar bone occurs [10,11]. Conventional corticotomy, piezocision, corticision, dentoalveolar distraction, accelerated osteogenic orthodontics, periodontal distraction, micro-osteoperforation (MOP), and interseptal alveolar surgery are among the surgical procedures used for accelerating tooth movement [8]. Among the surgical techniques, MOP, a minimally invasive technique, involves inducing small perforations in the alveolar bone using mini-screws or special instruments without raising a flap [12,13].

In comparison to a conventional corticotomy, an MOP is considered to be less invasive, convenient, and effective [14]. As stated by Frost [10], MOPs stimulate cell biodiversity and cause RAP. When subjected to a regional stimulant intervention like minor MOPs, the RAP is a process in which a repairing tissue recovers at a faster rate than the usual repair process. The biological response to orthodontic force plays a vital role in the manner and rate of tooth movement. The rate of tooth movement is closely related to bone resorption and osteoclastic activity [10,11]. In clinical settings, the application of MOP substantially boosts cytokine expression, resulting in a 60% reduction in treatment duration relative to a control group and 2.3 times faster canine retraction [8,15].

A greater understanding of the patient-reported outcome measures (PROMs) related to surgical treatments, including pain, discomfort, functional impairments, and satisfaction, has emerged with the widespread use of surgically-assisted acceleration of orthodontics [16,17]. A recent systematic review found limited evidence that these techniques cause mild to moderate pain and discomfort on the first day, but these symptoms disappear entirely within a week [17]. Most of the studies in the literature focus on canine retraction, with few studies examining maxillary molar distalization [12,17]. MOPs were applied buccally only in most studies except one [12], which compared buccal application to buccal and palatal application.

Therefore, the current study aimed to assess the pain levels during orthodontic treatment of class II malocclusion patients undergoing maxillary molar distalization assisted with different MOP approaches.

## Materials and methods

Trial design and ethical approval

The current study was a single-center parallel-arm randomized clinical trial with a 1:1:1 allocation ratio and superiority trial framework. This investigation was approved by the Ethics Committee, Faculty of Dental Medicine (Boys), Al-Azhar University, Cairo, Egypt (Approval number: 651/2053) and registered on ClinicalTrials.gov (ID: NCT05171738).

Participants 

Participants were selected randomly from the outpatient clinic of the Orthodontic Department, Faculty of Dental Medicine, Al-Azhar University (Boys). After explaining the study procedures to the eligible patients and signing informed consent, patients were enrolled in the study and randomized to receive repeated applications of MOP either on the buccal side only, both buccal and palatal sides, or in a control group (without MOPs). The selection criteria for the participants were as follows: adolescents (12 males and 18 females) with ages ranging from 14 to 17 years with bilateral class II molar relationship, skeletal class I or mild class II, normal or decreased vertical height, good oral hygiene, and fully erupted first and second molars. Participants were excluded if they had congenital dental-skeletal disorders or required surgical correction, posterior crowding or spacing, periodontally compromised teeth, and bad oral hygiene. Patients were discontinued from the trial if they experienced repeated appliance breakage and missed multiple appointments.

Sample size calculation

The sample size was calculated using G*power freeware (Release 3.1.9.7, 2020; Heinrich-Heine-Universität Düsseldorf, Düsseldorf, Germany) [18] with the following parameters: 80% power, a one-way ANOVA test for multiple means, and a 5% two-sided significance level. The estimated minimal sample needed to have adequate power to detect a clinical difference was 24. The total sample size was increased to 27 to account for possible drop-outs.

Randomization

Allocation sequence generation was done by computer-generated simple randomization using online software [19]. Allocation sequence concealment was done via telephone as the random number list was kept secured with the supervisor who was not involved in the procedures or the outcome assessment. After enrolling eligible participants, they were randomly assigned to either of the following three groups: (i) Group 1 comprising MOPs on the buccal surface, (ii) Group 2 comprising MOPs on the buccal and palatal surfaces, and (iii) Group 3, which was the control or no-MOP group. Blinding of the operators and the patients was not conceivable due to the nature of the intervention; only the statistician was blinded to the data analysis using codes assigned to different groups.

Interventions

**Figure 1:**
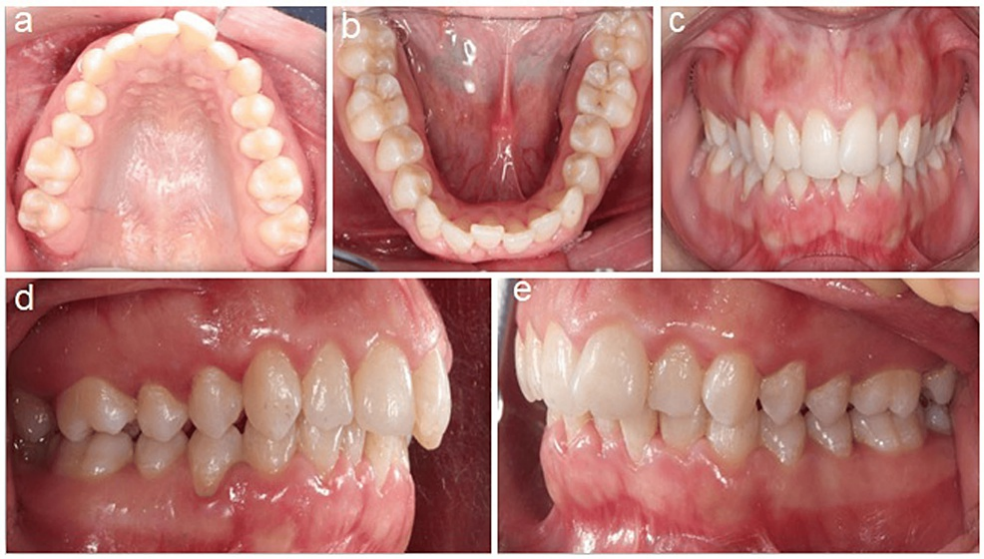
Pre-treatment intra-oral photographs of a 14-year-old female patient. a) Upper occlusal view, b) Lower occlusal view, c) Frontal view, d) Right lateral view, and e) Left lateral view

Maxillary molar distalization for all participants was performed through the distal jet appliance (American Orthodontics, Sheboygan, Wisconsin, United States). Separation and banding of the maxillary first molars and premolars were undertaken before impression taking and appliance fabrication. The appliance was fabricated as one unit with four solder joints at the first premolar and first molar bands. The mini-implant insertion slots were designed to be located 1 mm distal to the third rugae area, 3 mm lateral to the midpalatal raphe, and 3 mm away from the palatal mucosa [20]. Figure 1 presents the representative pre-treatment intra-oral photographs of a 14-year-old female patient.

**Figure 2:**
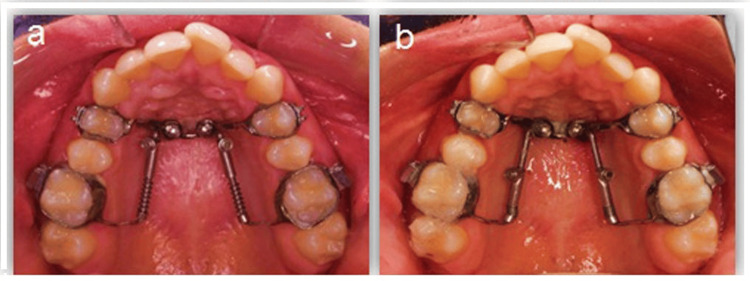
Intra-oral photographs of a female patient with distal-jet appliance and mini-implants in place. a) Immediately before distal jet activation and b) During distalization

After appliance insertion and band cementation, patients were given oral hygiene measures for two weeks before mini-implant placement as prophylaxis. After administration of local anesthesia and disinfection of the site, two mini-implants (OAS-T1511; BioMaterials Korea, Inc., Seoul, Republic of Korea) were installed into the 2 mm-diameter insertion slot to be perpendicular to the palate and directed away from the roots of the adjacent teeth [21]. Figure 2 presents the intra-oral photographs of a 14-year-old female patient during and after activation of the distal jet appliance with the mini-implants inserted in their slots.

**Figure 3:**
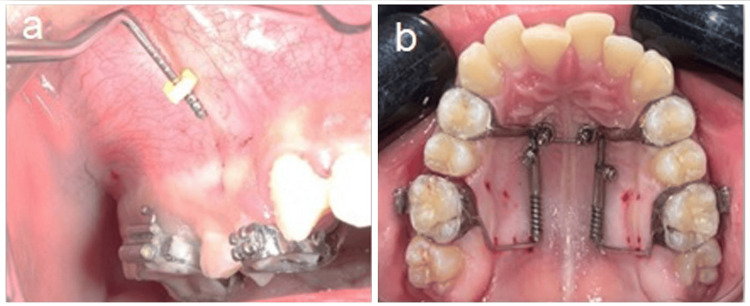
Application of micro-osteoperforations. a) Buccal and b) Palatal

Before the MOP application, patients were asked to rinse their mouths with 0.2% chlorhexidine mouthwash. Subjects in the MOP groups received MOPs in a repeated manner with each activation of the distalization appliance, where six MOPs were performed on the buccal side only or on both buccal and palatal sides with each activation of the appliance during the observation period of the study [22,23]. Under local anesthesia, two MOPs were applied between the second premolars and first molars, first molars, and second molars, and distal to the second molars (Figure 3) using 1.4 mm wide orthodontic mini-screws (Osstem Orthodontics, Inc., Gyeonggi-do, Republic of Korea). The MOPs were performed at a depth of 5-6 mm until the leading edge of the drill entered into the spongey bone by crossing through the cortical plate [24]. After the first activation, subjects were scheduled after four weeks for subsequent activation. After each MOP application, participants were instructed to use chlorhexidine mouthwash three times daily for three days and avoid non-steroidal anti-inflammatory drugs since they could hinder tooth movement [25].

**Figure 4:**
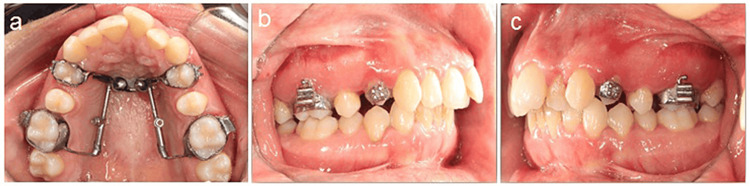
Post-distalization intra-oral photographs of a female patient. a) Upper occlusal view, b) Right lateral view, and c) Left lateral view

Figure 4 presents the post-distalization intra-oral photographs of a female patient. 

Outcomes 

Pain intensity was evaluated by a 10 cm visual analog scale (VAS) after activation of the distal jet appliance and MOP application. Pain intensity was measured 24, 48, 72 hours, and seven days after the first activation (T0) and the second activation (T1) after four weeks [26].

Statistical methods

All the study variables were collected, coded, and analyzed with the IBM SPSS Statistics for Windows, Version 23.0 (Released 2015; IBM Corp., Armonk, New York, United States). Kolmogorov-Smirnov and Shapiro-Wilk tests of normality tested the distribution of quantitative data. Data were statistically described in terms of means and standard deviations. A one-way ANOVA test was used to compare means from the three different groups at different time points, while the repeated measures ANOVA test was used to compare means in each group between different time points. The level of statistical significance was set at 5%. 

## Results

**Figure 5:**
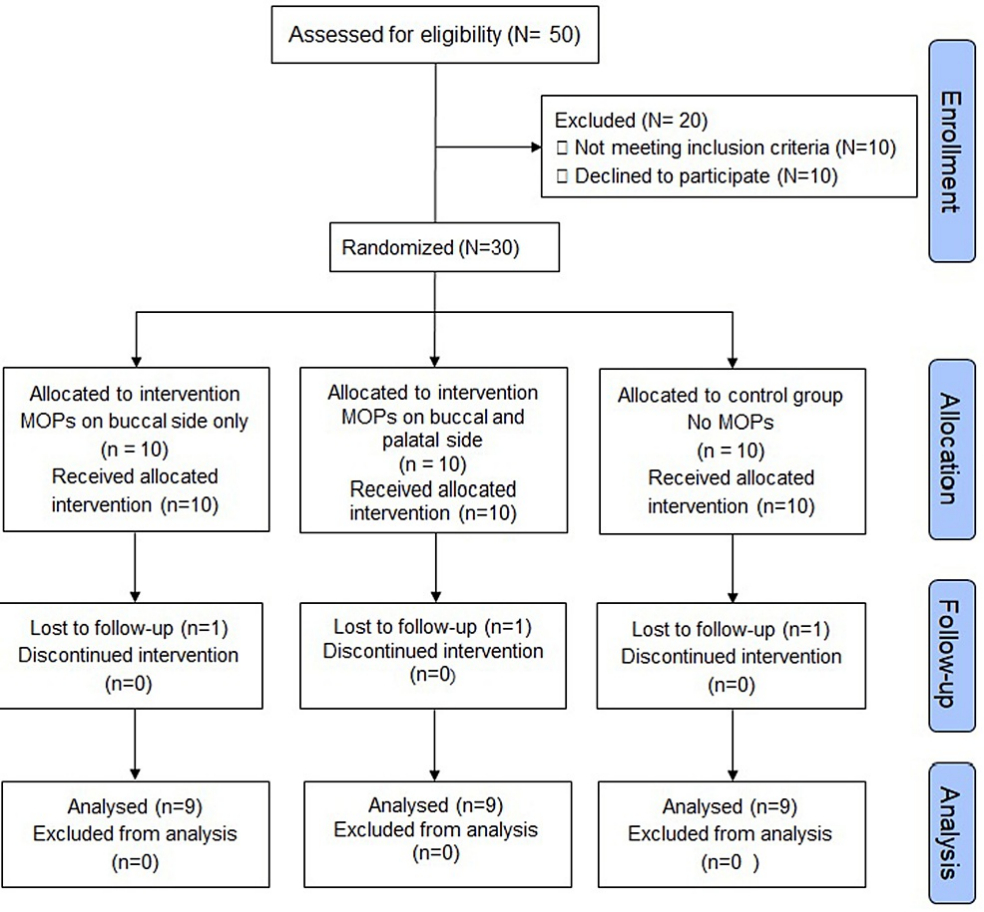
CONSORT flowchart CONSORT: Consolidated Standards of Reporting Trials; MOP: micro-osteoperforation

Figure 5 presents the Consolidated Standards of Reporting Trials (CONSORT) flowchart showing the study process. Out of the 50 participants, 10 participants did not meet the inclusion criteria and 10 participants declined to participate. Therefore, 30 participants who fulfilled the inclusion criteria with ages ranging from 14 to 17 years were enrolled and assigned randomly to one of the three groups (n=10) to receive MOPs after activation of the distalization appliance. However, one participant in each group was lost to follow-up and the remaining nine participants in each group were available for the final analysis. 

Table 1 shows the baseline characteristics of the study participants. The mean age of the patients in Group 1, Group 2, and Group 3 were 15.5 ± 0.3, 16.5 ± 0.4, and 16 ± 0.5 years, respectively. Among the enrolled patients, nine were males and 18 were females. 

**Table 1:** Baseline characteristics Group 1: MOPs on the buccal side only; Group 2: MOPs on the buccal and palatal side; Group 3: No MOPs (control); M: males; F: females; SD: standard deviation.

Variables / Groups	Group 1 (n=9)	Group 2 (n=9)	Group 3 (n=9)
Gender (M/F)	2/7	3/6	4/5
Age (years), mean ±SD	15.5 ± 0.3	16.5 ±0.4	16 ± 0.5

Table 2 shows intergroup and intragroup comparisons among treatments at different time points for the first and second activation. After the first activation, the MOP groups (Group 1 (4.8 ± 2.1) and Group 2(5.5 ± 2.3)) demonstrated the highest pain intensity compared to the control group with no MOPs (2.6 ± 2.2) after 24 hours of activation. The difference in pain intensity between the groups after 24 hours of activation was statistically significant (p=0.003). However, there was no significant difference in the pain intensity among the groups at 48 hours (p=0.451), 72 hours (p=0.781), and seven days (p=0.842). The intragroup comparison of the pain intensity at different time points demonstrated that pain intensity decreased significantly in Group 1 (p=0.004) and Group 2 (p=0.008), whereas no significant decrease was observed in Group 3 (p=0.356). 

**Table 2:** Intergroup and intragroup comparison of treatments at different time Group 1: MOPs on the buccal side only; Group 2: MOPs on the buccal and palatal side; Group 3: No MOPs (control) *statistically significant (p≤ 0.01).

Timepoint/Group	Group 1 (n=9)	Group 2 (n=9)	Group 3 (n=9)	p-value
T0 (1^st^ activation)				
24 hours	4.8 ± 2.1	5.5 ± 2.3	2.6 ± 2.2	0.003*
48 hours	3.1 ± 2.2	3.4 ± 3.2	2.1 ± 1.9	0.451
72 hours	1.6 ± 2.9	1.9 ± 3.1	1.8 ± 3.2	0.781
7 days	0.8 ± 1.5	0.9 ± 1.2	0.9 ± 1.4	0.842
p value	0.004*	0.008*	0.356	
T1 (2^nd^ activation after 4 weeks)				
24 hours	2.3 ± 1.4	2.5 ± 1.9	1.6 ± 1.4	0.568
48 hours	1.7 ± 0.6	1.9 ± 0.5	1.4 ± 0.6	0.072
72 hours	0.4 ± 0.5	0.3 ± 0.4	0.8 ± 0.7	0.845
7 days	0.1 ± 0.2	0.2 ± 0.1	0.4 ± 0.3	0.914
p-value	0.021*	0.031*	0.641	

At 24 hours of second activation, Group 3 demonstrated lowest pain intensity (1.6 ± 1.4) followed by Group 1 (2.3 ± 1.4) and Group 3 (2.5 ± 1.9). However, the difference in pain intensity between the groups was statistically non-significant (p=0.568). Similarly, the comparison of pain intensity between the groups at 48 hours (p=0.072), 72 hours (p=0.845), and seven days (p=0.914) was non-significant. The intragroup comparison of the pain intensity at different time points demonstrated that pain intensity decreased significantly in Group 1 (p=0.021) and Group 2 (p=0.031), whereas the pain decrease was not significant in Group 3 (p=0.356). 

## Discussion

This study aims to measure pain levels experienced by patients with Class II malocclusion undergoing maxillary molar distalization assisted with MOPs as a means of non-invasive, surgically-assisted acceleration of orthodontic tooth movement. This study is the first randomized control trial with a multi-arm design that analyzes the efficacy of different MOP applications to the buccal side or both the buccal and palatal sides with a maxillary molar distalization appliance in the context of recent systematic reviews [12,17]. All the patients received the same distalization appliance. According to a standardized protocol, they were randomized to receive MOPs on the buccal side or buccal and palatal or none after appliance activation. Pain intensity was measured using a 10 cm VAS after each activation at 24 hours, 48 hours, 72 hours, and seven days, as recommended by a recent systematic review on PROMs regarding the effectiveness of surgically assisted acceleration of orthodontic treatment [17].

The contemporary results indicated that pain intensity was significantly higher in the MOP groups compared to the control group. The highest pain values were observed after the first activation of MOPs applied to both alveolar sides. The pain levels varied from mild to moderate on the first day and dropped to mild in the following days until virtually none after seven days. This result agrees with a study by Gulduren et al., which showed higher pain intensity levels on the first day for the MOP group in maxillary molar distalization [24]. However, Gulduren et al.'s study only applied MOPs on the buccal side. A study by Babanouri et al. reported no weighty pain intensity when using MOPs applied on buccal or buccal and palatal sides in canine retraction [22]. Pain intensity decreased considerably between the first and second activations and between different time points in the MOP groups. This concurs with the study by Gulduren et al. [24].

Previous studies utilizing MOPs showed mild to moderate pain levels in the interventional groups [12,17]. Over time, the pain decreased to almost equal for the control groups, consistent with the current findings. There is a disagreement in the pain intensity values among various studies, as revealed by a recent systematic review [11]. This disagreement could be attributed to the different study designs used, which were split-mouth design and the application of MOPs in situations other than maxillary molars distalization. The use of split-mouth designs could confuse the patient, as they may experience pain on only one side where the MOPs were applied, which is a significant difference from our trial design that could be more beneficial in showing the effect of bilateral application of MOPs compared to a control group [17].

The MOP groups experienced higher levels of pain compared to the control one due to the trauma and injury to the gingival tissues, periosteum, and cortical bone, as well as the pain caused by the orthodontic activation [17]. Investigations suggest that bone injury triggers the release of cytokines, resulting in an increase in bone turnover and a decrease in regional bone density. These cytokines/chemokines initiate and maintain pathological pain by directly activating nociceptive sensory neurons [27,28].

Regarding the PROMs with the use of orthodontic acceleration techniques, previous studies have reported varying outcomes. Khlef et al. compared the PROMs between patients treated with flapless corticotomy versus traditional corticotomy [16]. After 24 hours of surgery, traditional corticotomies were accompanied by mild to moderate pain, difficulties in swallowing, moderate discomfort, trouble chewing, and limitation in jaw movement. On the contrary, flapless corticotomy presented with fewer complications and was characterized by mild pain, swelling, trouble chewing, limiting jaw movement, and difficulties in swallowing throughout the same assessment period. Flapless corticotomy was associated with higher levels of patient satisfaction, acceptance, and friend referral than standard treatments. Alfailany et al. compared the PROMs with the use of low-level laser therapy (LLLT)-assisted canine retraction versus Piezocision™-assisted retraction versus conventional canine retraction [29]. Compared to Piezocision-assisted retraction, LLLT-assisted canine retraction was demonstrated to have noticeably fewer adverse patient-reported outcomes throughout the first two weeks of retraction. During the first week of retraction, however, the degrees of pain and suffering were significantly higher in the group receiving Piezocision-assisted retraction than in the traditional canine retraction group, which was higher still than in the LLLT-assisted canine retraction group. Both the LLLT and Piezocision procedures demonstrated great levels of patient acceptability and satisfaction.

It is important to note that the current trial had certain limitations, including assessing pain for only a short period (four weeks) during the entire process of maxillary distalization. Secondly, the thickness of buccal and palatal bone thickness could affect the pain outcome, which was not considered in this study. Finally, the sample size was relatively small without gender difference, and the patients enrolled were young. At this age, tooth movement tends to be quicker due to red bone marrow and greater bone remodeling capacity. On the other hand, in older patients, the effects of MOP might be more significant [30].

The current study has several strengths: It used a multi-arm parallel design, which allowed for a comparison between applying MOPs on the buccal or buccal and palatal sides bilaterally. It included a control group that did not receive MOPs, making the results more reliable. It is recommended to conduct more high-quality, more extensive, randomized clinical trials to assess the effect of MOPs on pain and patient-reported outcomes. It would be interesting to include adult or older patients in future clinical trials and compare the outcome with that of the young or adolescent patients.

## Conclusions

The application of MOPs as a non-invasive, surgically-assisted orthodontic acceleration technique for maxillary molars distalization resulted in mild to moderate pain intensity levels. The pain levels were unaffected by the repeated application of MOPs on the buccal or both the buccal and palatal sides. Moreover, the pain gradually decreased to mild levels in the subsequent days.
